# Preclinical study of dimebon on β-amyloid-mediated neuropathology in Alzheimer's disease

**DOI:** 10.1186/1750-1326-6-7

**Published:** 2011-01-19

**Authors:** Jun Wang, Mario G Ferruzzi, Merina Varghese, Xianjuan Qian, Alice Cheng, Mathew Xie, Wei Zhao, Lap Ho, Giulio M Pasinetti

**Affiliations:** 1 Department of Neurology, Mount Sinai School of Medicine, New York, New York 10029 USA; 2 Department of Psychiatry, Mount Sinai School of Medicine, New York, New York 10029 USA; 3 Departments of Food Science and Foods&Nutrition, Purdue University, West Lafayette, Indiana 47907 USA; 4 Department of Neuroscience, Mount Sinai School of Medicine, New York, New York 10029 USA; 5 Geriatric Research, Education and Clinical Center, James J. Peters Veterans Affairs Medical Center, Bronx, New York 10468 USA

## Abstract

**Background:**

Dimebon is a retired non-selective antihistamine drug currently being investigated as a therapeutic agent for the treatment of Alzheimer's disease (AD). Results from several completed clinical trials are mixed and contradictory. Proper interpretations of these clinical observations, as well as future development of dimebon in AD treatment are complicated by the lack of concrete information on the mechanisms by which dimebon might benefit AD.

**Results:**

The present studies are designed specifically to assess whether dimebon might modulate β-amyloid (Aβ)-mediated responses which are central to the development and progression of AD dementia. We found that dimebon is bioavailable in the brains of mice following oral administration. AD mice chronically treated with dimebon exhibited a trend of improvement in spatial memory function without affecting the levels of total Aβ as well as soluble oligomeric Aβ in the brain. The same trend of behavior improvement is also seen in wild type animals chronically treated with dimebon.

**Conclusion:**

Collectively, our preclinical studies using the TgCRND8 AD mouse model demonstrated that dimebon might have some beneficial effect in improving cognitive function independent of Alzheimer's disease-type Aβ-related mechanisms or global energy metabolism in the brain. Observations from our study and others suggesting dimebon might improve cognition in wild type mice and rats raises the possibility that dimebon might be able to benefit cognitive function in patients with other neurodegenerative disorders, such as Huntington's disease, or in the aging population. Additional studies will be necessary to clarify the mechanisms by which dimebon might directly or indirectly benefit cognitive function.

## Background

The drug latrepirdine (Dimebon ^® ^or Dimebolin) is an old non-selective antihistamine drug that showed clinical benefits in an eight week, open-label, pilot study with 14 Alzheimer's disease (AD) patients in Russia [1]. In a subsequent double-blind, placebo-controlled phase II clinical trial held in Russia, dimebon significantly improved and stabilized cognitive function in mild-to-moderate AD patients over a one-year period [2] and continued to preserve all key functions when the clinical trial was extended to a six months open-label extension or 18 months into the trial. However, in the most recent multi-national, double-blind, placebo-controlled confirmatory phase III clinical trials, dimebon failed to show statistical improvements relative to placebo in cognition and global function in patients with mild-to-moderate Alzheimer's disease (AD). This drastic difference was unexpected and very difficult to decipher, especially in view of the fact that no preclinical information on the physiological activities of dimebon on AD-related mechanisms and phenotypes *in vivo *are available.

Several dimebon clinical trials are still ongoing, including a phase III clinical research study (the CONCERT Study) designed to evaluate whether the application of dimebon in combination with Aricept® (donepezil HCl) can improve cognition and daily living function in 1050 patients with mild-to-severe AD. Another ongoing clinical trial is designed to evaluate the safety and efficacy of dimebon in patients with moderate-to-severe AD receiving existing background therapy with memantine. In addition to AD, dimebon is also being developed for treating Huntington's disease (HD). An earlier phase II study showed that dimebon treatment led to a modest, but significant improvement in cognitive functions in HD patients as measured by the Mini Mental States Exam in HD patients [3]. A multinational phase III Huntington disease trial (the HORIZON Study) is currently in progress to test the safety and efficacy of dimebon in HD patients.

AD is a multi-faceted disease and understanding the mechanism(s) of action of any potential treatments is of great importance. The aggregation of β - amyloid (Aβ) peptides into high molecular weight neurotoxic species and subsequent formation of amyloid plaques is one of the two defining neuropathological hallmark of AD. Most of the current therapeutic strategies are designed to target Aβ by interfering with the synthesis or degradation of Aβ, or by interfering with aggregation of Aβ into neurotoxic high molecular weight aggregates. The present study was designed to evaluate the bioavailability of dimebon in the brain and to explore the preclinical efficacy of dimebon in modulating Aβ-mediated mechanisms using experimental mouse models of AD. Our study may provide important information in understanding the results from the clinical trials for dimebon in AD. Moreover, the current study will also provide insights for future application of this drug in AD as well as in HD or other neurological disorders.

## Materials and methods

### Dimebon

Dimebon was acquired from Ryan Scientific, Inc (Mt. Pleasant, SC). In initial quality control studies, the 1D NMR proton spectra of dimebon acquired from Ryan Scientific, Inc. is comparable to the spectra for dimebon published by Wu et al. [4]. The purity of the preparation is 99% (data not shown).

### Bioavailability studies

Wild type mice (strain-, age-, gender-matched with TgCRND8 mice used for preclinical efficacy study described below) were used for dimebon bioavailability studies. Two months old mice were chronically treated with 12 mg/kg/day dimebon for 4 months delivered through their drinking water. On the day of testing, mice were gavaged with the daily dose of dimebon and plasma and brain samples were collected at 0, 30, 60, 120 minutes following gavage. Extraction of plasma and brain tissues was accomplished using the method described by Nirogi et al. [5] with modification. Briefly, 250 μ L of plasma was extracted with 3.0 mL of hexane:tetrahydrofuran (THF) (20:80). Samples were vortexed for 30 s, after which the organic layers were collected. Samples were extracted a total of 3 times and combined organic layers were dried under vacuum and analyzed immediately. Brain tissues (~400 mg) were carefully weighed, placed in 3.0 mL of phosphate buffered saline (pH = 7.4) and sonicated to produce a homogenate. Brain homogenates were then extracted following the method for plasma described above.

### LC-MS analysis of dimebon

Analysis of dimebon in plasma and brain extracts was accomplished by LC-MS using a system consisting of a Waters model 2795 separations module equipped with a ZQ2000 mass selective detector (MSD) (Waters Corp. Milford, MA). Dried extracts were dissolved in 200 μL of 20:80 methanol: ddH_2_O and 20 μL was injected onto the LC-MS system. Dimebon was resolved within 5 minutes using a reversed-phase Xterra C_18 _column (100 × 2.1 mm i.d.) (Waters Corp, Milford, MA) by isocratic elution with ddH_2_O:acetonitrile:formic acid (64.9:35:0.1) at a flow rate of 0.2 mL/min. Following separation the column effluent was introduced by positive mode electrospray ionization (ESI) into a Waters ZQ MSD. ESI capillary voltage was 3.5 kV, and the source and desolvation temperatures were 150°C and 350°C, respectively. Selected Ion Response (SIR) detection was performed for m/z 320 with a span of ± 0.50 to detect dimebon. Chromatographic and spectroscopic data was collected and analyzed using Empower2 software.

Quantification of dimebon was accomplished using calibration plots constructed from authentic dimebon standard reference material (Ryan Scientific). A stock solution of dimebon was prepared in 20:80 methanol:ddH_2 _O at 500 μM. The stock solution was volumetrically diluted in series with methanol:ddH_2 _O to between 0.01-100 nmol/mL. 20 μL aliquots of these solutions were injected onto the LC-MS and calibration plots constructed by plotting peak area versus dimebon concentration.

### AD mice and treatment

Female TgCRND8 AD transgenic mice expressing human amyloid precursor protein (APP) containing Indiana and Swedish double mutation (KM670/671NL+V717F) were used in the preclinical study. TgCRND8 mice are characterized by an early onset of AD phenotype including memory impairment at 3-months of age [6]. Both female TgCRND8 mice and WT female littermates were housed with food and water available ad libitum, and maintained on a 12:12 h light/dark cycle with lights on at 07:00 h in a temperature-controlled (20 ± 2°C) room prior to experimental manipulation. All procedures and protocols were approved by the Mount Sinai School of Medicine's Institutional Animal Care and Use Committee (IACUC) through the Center for Comparative Medicine and Surgery. Mice were randomly divided into two groups, the non-treated control group (CTRL) and the dimebon-treated group (dimebon). The treatment started at 2 months of age prior to the onset of AD-type neuropathology. At 2 months of age, the amyloid load in TgCRND8 mice is about *100 ng/g tissue for*Aβ_1-42_*and 56 ng/g tissue for*Aβ_1-40 _[6]. The animals were treated for 4 months till the animals reached 6 months of age. In this specific mouse model of AD, the amyloid neuropathology was very aggressive and the amyloid content and plaque load at 6 months of age are equivalent to those found in old Tg2576 mice [7,8]. Animals were treated with 12 mg/kg/day dimebon, the dose equivalent to the human dose of 20 mg t.i.d used in the clinical trial using FDA criteria for converting drug equivalent dosages across species, based on body surface area ([human equivalent dose in mg/kg = animal dose in mg/kg × (animal weight in kg/human weight in kg)^0.33^] (http://www.fda.gov)].

### Behavioral assessment of cognitive function by the Morris water maze test

Spatial learning memory was assessed by the Morris water maze (MWM) behavioral test, as previously described [9,10]. Mice were tested in a circular pool filled with water mixed with non-toxic white paint (Dick Blick Art Materials, IL). In the initial learning phase, mice were trained for 7 or 8 consecutive days to allow them to learn to escape from the water onto a hidden/submerged (1.5 cm below-water surface) escape platform (14 × 14 cm) in a restricted region of the pool using the spatial cues provided. Spatial learning memory was assessed by recording the latency time for the animal to escape from the water onto escape platform as a function of the number of learning trials during the learning phase. Twenty-four hours after the last learning session, mice were subjected to a 45 second probe trial wherein the escape platform was removed. Spatial memory retention is reflected by the percentage of time animals spent within the "target" quadrant of the pool that previously contained the hidden escape platform. Water maze activity during training and probe trials was monitored with the Poly-Track video tracking system (San Diego Instrument).

### Assessment of AD-type amyloid neuropathology

Total Aβ_1-40_or Aβ_1-42_in the brain were quantified by sandwich ELISA (Invitrogen, Carlsbad, CA), as previously described [11]. The level of soluble Aβ oligomers was measured by a commercially available sandwich ELISA (Invitrogen, Carlsbad, CA) according to the manufacturer's instruction. Specifically, soluble amyloid peptide was extracted in PBS and centrifuged at 78,500 × g for 1 h at 4°C, and the supernatant was quantified by ELISA to specifically detect aggregated Aβ [12]. According to the manufacturer, the same monoclonal antibody specific for the N-terminus of human Aβ were used both as capturing and detecting antibody.

### Cell cultures and dimebon treatment

Embryonic-day (E)15 cortico-hippocampal neuronal cultures were prepared from heterozygous TgCRND8 transgenic mice (TgCRND8 neurons) as previously described [10]. Neurons were treated with different concentration of dimebon in triplicate for ~16 hours and conditioned medium was collected for Aβ detection using commercially available kits (Invitrogen, Carlsbad, CA).

### In vitro Aβ_1-42 _aggregation assay

Aβ_1-42_peptides were purchased from American Peptide (Sunnyvale, CA). Peptides were solublized in 1,1,1,3,3,3,-hexafluoro-2-propanol (HFIP) from Sigma, and dried overnight. Dimebon was dissolved in phosphate buffer (pH 7.4), mixed with Aβ_1-42 _at 1:1 molar ratio and incubated at 37°C for 24 hours. The effect of dimebon on Aβ aggregation was analyzed by western blot analysis using 6E10 antibody.

### Assays for tricarboxylic acid (TCA) cycle enzymes activities and protein levels

TgCRND8 mice treated with dimebon or vehicle were sacrificed and their brains were rapidly dissected. The brains were frozen at - 80°C till the day of the assays. The tissue was homogenized in sucrose buffer (0.32 M sucrose, 0.125 M Tris, 0.1 mM EDTA, 0.6 mM MgCl_2_, 1 mM DTT, with protease inhibitors, pH 8.0) using Precellys 24 (Bertin Technologies, Montigny-le-Bretonneux, France) at 6500 rpm for 8 s. The homogenate was centrifuged, assayed for protein by the method of Bradford (1976) and immediately used to measure enzyme activities.

Alpha-ketoglutarate dehydrogenase complex (KGDHC, oxoglutarate dehydrogenase, EC 1.2.4.2) activity was measured as per Gibson et al (1988)[13]. The reaction mixture (200 μl) contained assay buffer (50 mM Tris pH 7.0, 1 mM MgCl_2_, 1 mM CaCl_2_, 0.5 mM K-EDTA, 1 mM dithiothreitol, 1% triton X-100), 0.3 mM thiamine pyrophosphate, 1 mM NAD, 0.163 mM coenzyme A and the sample (25-30 μg protein). After the baseline stabilized at 37°C (about 5 min), 1.25 mM 2-ketoglutarate was added to the reaction mixture and the formation of NADH was monitored for 5 min at 340 nm using a VERSAmax microplate reader and SoftMax Pro 5.3 software (Molecular Devices, Sunnyvale, CA). The activity was expressed as nmol NAD^+ ^oxidized/min/mg protein (ε_340 _= 6.23 mM^-1 ^cm^-1^).

The malate dehydrogenase (MDH, EC 1.1.1.37) activity assay was modified from the method of Rokosh et al (1973)[14]. For the MDH assay, the 200 μl reaction mix contained 50 mM sodium glycine buffer, 30 mM malate, 10 μM rotenone, 1 mM NAD^+^, 0.055 mM phenazine methosulfate, tissue homogenate (3-10 μg protein) and 0.11 mM 2, 6-dichlorophenolindophenol (DCPIP) Following the baseline reading for 1 min, the reaction was initiated by adding DCPIP and absorbance was measured at 600 nm for 1 min at 37°C. The activity was expressed as nmol DCPIP reduced/min/mg protein (ε_600 _= 19.1 mM^-1^cm^-1^).

Citrate synthase (Citrate oxaloacetate-lyase (CoA-acetylating), E.C.4.1.3.7) activity was measured using the citrate synthase assay kit (Sigma-Aldrich, MO, USA) following the manufacturer's instructions with 5-15 μg of protein in each reaction. The activity was expressed as nmol 5, 5'-dithiobis (2-nitrobenzoic acid) (DTNB) reduced/min/mg protein (ε_412 _= 13.6 mM^-1^cm^-1^).

The same lysate was used for western blot analysis to measure the protein levels of these enzymes following dimebon treatment. Sheep anti-MDH (Rockland Immunochemicals, PA), goat anti-citrate synthase (Santa Cruz, CA) and goat anti-alpha-KGD (Santa Cruz, CA) were used to evaluate the levels of these proteins by western blot analysis and immunoreactive signals were quantified densitometrically.

### Statistical analysis

All values are expressed as mean and standard error of the mean (SEM). Differences between means were analyzed using either 1 - way or 2-way repeated measures ANOVA or 2-tailed Student *t *test. In all analyses, the null hypothesis was rejected at the 0.05 level. All statistical analyses were performed using the Prism Stat program (GraphPad Software, Inc., San Diego CA).

## Results

### Dimebon is bioavailable and can be detected in the plasma and brain

**Figure 1:**
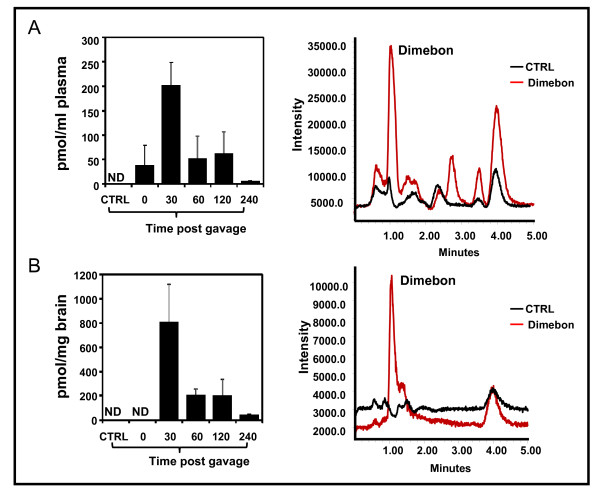
**Plasma and brain pharmacokinetics of dimebon following acute on chronic dosing in wild type mice**. (A) Plasma pharmacokinetic profile of dimebon following acute on chronic dosing of dimebon (12 mg/kg BW) over 6 hours, right panel: LC-MS separation of dimebon detected in extracts of plasma (B) Brain pharmacokinetic profile of dimebon following acute on chronic dosing of dimebon (12 mg/kg BW), right panel: representative LC-MS separation of dimebon detected in extracts of mouse brain tissues. Data represents mean ± SD n = 3 mice per group.

Bioavailability studies were conducted by measuring plasma pharmacokinetic response (0-6 h) of dimebon in mice following chronic dimebon treatment. Following chronic exposure, we tested plasma pharmacokinetic response to a single acute dose of dimebon. Six-hour plasma pharmacokinetic profile of dimebon in mouse plasma is presented in Figure 1A, left panel. Following the oral dose of dimebon, plasma levels of dimebon peaked within 30 minutes, reaching a maximum concentration of 202.17 ± 46.14 pmol/mL of plasma. Elimination of dimebon was rapid as observed by a significant drop in plasma levels between 30 minutes and 60 minutes with levels returning to baseline levels by 6 h following dimebon administration. There are two additional peaks besides dimebon, which appeared in the LC-MS separation profile in plasma from dimebon treated animals, but not from control vehicle gavaged animals (Figure 1A, right panel), suggesting potential formation of metabolites. Additional studies are required to elucidate the potential for dimebon metabolism.

Following plasma pharmacokinetics of dimebon, we continued to explore whether dimebon might be accumulated in the brain following chronic treatment. As we have done in our plasma pharmacokinetic studies, mice were chronically treated with dimebon. Thereafter, mice were given a single, daily dose of dimebon. Brains were isolated at different time points extracted and analyzed by LC-MS as described previously. Similar to our observations in the plasma, we found dimebon levels in the brain to peak within 30 minutes following the oral dose of dimebon, reaching 810.22 pmol/mg brain. Similar to plasma pharmacokinetics, elimination of dimebon in the brain was also very rapid and a significant drop was observed between 30 minutes to 60 minutes with levels returning to baseline by 6 hours (figure 1B, left panel). Interestingly, no additional peak was seen in the brain LC-MS profile, indicating that very likely only dimebon but no additional metabolites, reaches the brain (Figure 1B, right panel).

### Dimebon may have a positive effect on cognition both in TgCRND8 and strain-, age-, gender - matched wild type mice

**Figure 2:**
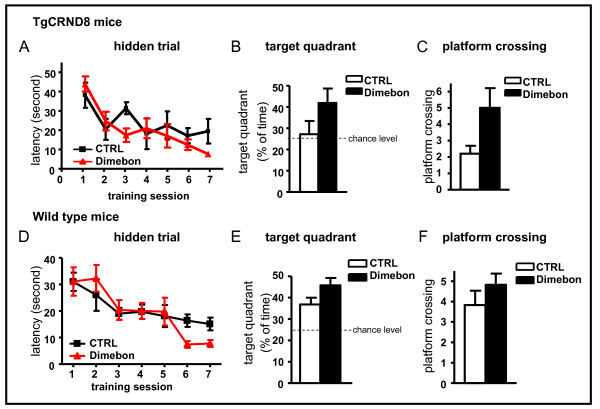
**The influence of chronic dimebolin treatment on spatial memory in TgCRND8 and wild type mice in the Morris water maze test**. (A, D) Hidden platform acquisition from TgCRND8 mice (A) and strain-, age-, gender - matched wild type mice (D). Latency score represents the time taken to escape to the platform from the water. (B, E) Probe trials for TgCRND8 mice (B) and wild type mice (E) expressed as percentage of time spent in the target quadrant. Percent of time in target quadrant is calculated as the ratio of time spent in the target quadrant area relative to the time spent in the rest of the pool; (C, F) Probe trials for TgCRND8 mice (C) and wild type mice (F) expressed as platform crossing. Platform crossing is calculated as the times the animals cross the target platform.

TgCRND8 mice were treated with 12 mg/kg/day (equivalent to the human dose of 20 mg t.i.d used in the clinical trial), starting at 8 weeks of age. We found that 4-month dimebon treatment was well-tolerated by TgCRND8 mice, as reflected by their general health indexes such as normal food and water intakes, normal body weight and normal grooming of the mice (data not shown). One-way ANOVA of escape latencies during 7 days of water maze acquisition revealed that the dimebon-treated group improved significantly with each training session as reflected by a progressive reduction of escape latency over the 7-day training (Figure 2A, One-way ANOVA repeated measure; P < 0.0001). The none-treated control TgCRND8 group did not show significant difference during the 7-day training (One-way ANOVA repeated measure; p = 0.0704). However, the difference between the two groups did not reach statistical significance (two-way ANOVA repeated measure; p > 0.05) (Figure 2A). The dimebon-treated group had a trend of performing better than the control group during the probe trial 24 hours after the last training session, which is reflected by both the percentage of time the mice spent in the target quadrant and by the times the mice crossed the target platform (Figure 2B and 2C). However, neither reached statistical significance (p = 0.078 for platform crossing, p = 0.15 for quadrant occupation). These results indicate that dimebon treatment might help to consolidate spatial information and long lasting reference memory in this mouse model of AD.

A parallel control study with strain-, age - and gender - matched wild type mice showed that both treated and non-treated group showed a significantly reduced latency during the 7-day training (Figure 2D, one-way ANOVA repeated measure; P < 0.05 for the non-treated control group and p < 0.0001 for dimebon-treated group). Both groups performed well in the probe trial, as reflected by the percentage of time spent in the target quadrant well above the 25% chance level (Figure 2E). The dimebon-treated group performed slight better, however, did not reach statistical significance (p = 0.07).

All groups performed equally well in a visible trial, excluding the possibility that dimebon treatments might have affected non-spatial parameters such as sensorimotor performance and motivation (data not shown) which might have interfered with the behavioral performance during the MWM test.

### Dimebon treatment has no effect on amyloid accumulation in the brain of TgCRND8 mice or in primary neuron culture

**Figure 3:**
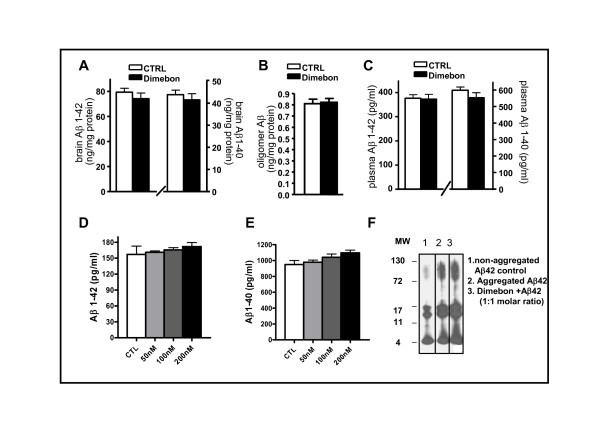
**The *in vivo *effect of dimebon treatment on Aβ-mediated neuropathology in TgCRND8 mice and the *in vitro *effect of dimebon on Aβ generation and aggregation**. (A) Total Aβ_1-40 _and Aβ_1-42 _content in the brain (B) Oligomeric Aβ content in the brain (C) Plasma level of Aβ_1-40 _and Aβ_1-42 _from TgCRND8 mice treated with 12 mg/kg/day dimebon for 4 months. (D) and (E) Secreted Aβ_1-42 _and Aβ_1-40 _levels in conditioned media from primary neuron culture derived from E15 APP transgenic mice following 20 hours dimebon treatment (dimebon concentrations: 0, 50 nM, 100 nM and 200 nM). (F) The effect of dimebon on Aβ_1-42 _aggregation *in vitro*.

Following the MWM behavior test, mice were sacrificed and brain Aβ levels were measured. We did not find any detectable changes in the content of total amyloid peptides (Aβ_1-42 _and Aβ_1-40_) in the brain of dimebon treated mice compared to non-treated control mice, nor did we find any changes in the level of extracellular high molecular weight oligomeric Aβ species, which are considered to be the main molecules contributing to cognitive impairments (Figures 3A and 3B), following 4 months of dimebon treatment. There was no change of plasma levels of Aβ_1-40 _or Aβ_1-42 _in TgCRND8 mice following dimebon treatment (Figure 3C). In parallel studies, we evaluated the effect of dimebon on APP processing using primary neuron culture derived from E15 APP transgenic mice. Consistent with the *in vivo *findings, we found that 20-hour treatment of primary neuron culture with various concentrations of dimebon does not lead to any significant changes in Aβ_1-42 _and Aβ_1-40 _contents in the conditioned medium (Figures 3D and 3E). Moreover, using an *in vitro *aggregation assay [15], we found that dimebon did not have any significant effect on Aβ_1-42 _peptides self-aggregation (Figure 3F). Collectively, results from our *in vitro *and *in vivo *studies exclude the possibility that APP processing as a direct target of dimebon.

### Dimebon treatment does not affect the activities of key metabolic enzymes in the brain of TgCRMD8 mice

**Figure 4:**
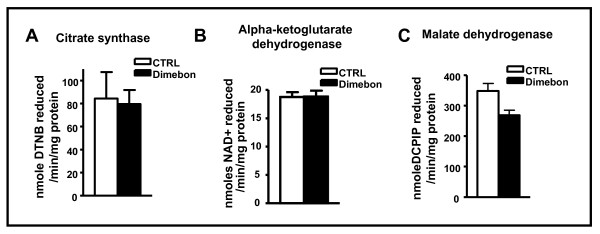
**The effect of dimebon on mitochondrial function in the brain of TgCRND8 mice**. The enzyme activities of (A) citrate synthase (B) alpha ketoglutarate dehydrogenase and (C) malate dehydrogenase in brain of TgCRND8 mice chronically treated with dimebon compared to non-treated control TgCRND8 mice.

It was previously proposed that one of the mechanisms underlying dimebon benefits is its ability to protect/preserve mitochondrial function in neurons upon beta amyloid assaults. To evaluate the impact of dimebon treatment on mitochondrial energy metabolic functions, we assessed whether long-term dimebon exposure might modulate citrate synthase, which is the key regulatory enzyme for the tricarboxylic acid cycle [16]. We found that dimebon treatment did not change the activity of citrate synthase in the brain (figure 4A), suggesting that, contrary to previous assertions, dimebon does not improve mitochondrial energy metabolism in the brain of TgCRND8 mice. This is also supported by our observation that dimebon treatment did not change the level of citrate synthase protein level in the brain (100 ± 24.7% in control mice vs. 94.2 ± 22.8% in dimebon treated mice). We also assessed two other enzymes that also contribute to the regulation of the Tricarboxylic Acid (TCA) cycle: alpha ketodehydrogenase complex (a-KGDH) and mitochondrial malate dehydrogenase. We found no detectable changes in the activity or protein level of a-KGDH in the brain following dimebon treatment (Figure 4B). Interestingly, we observed a ~25% reduction in the activity of mitochondrial malate dehydrogenase in the brain following dimebon treatment (Figure 4C). In view of the fact that citrate synthase is generally considered to be the key regulatory, "bottle-neck" enzyme for the TCA cycle, it is not clear whether the small reduction in mitochondrial malate dehydrogenase activity might lead to appreciable inhibition of mitochondrial energy metabolism. Collectively, our evidence suggests dimebon might not neuroprotect by improving mitochondrial energy metabolic functions.

## Discussion

Alzheimer's disease is a progressive neurological disorder currently affecting 5 million people in the United States [17]. Its prevalence increases exponentially with age and poses tremendous burden on public health. Treatment currently available can only temporarily stabilize or slow the progression of dementia and no effective cure exists for this devastating disease.

The success of a phase II trials of dimebon with small cohorts of AD patients brought huge excitement to the AD community. However, the subsequent failure of a much larger and better controlled phase III trials has left us with more questions than answers.

There is no preclinical information on whether dimebon might modulate AD related mechanisms or the basic science underlying its potential function in AD. Evidence from *in vitro *studies suggests dimebon might protect against Aβ-mediated toxicity in primary neuron cultures and might improve mitochondrial function in cultured cells in the presence or absence of stress [18-20]. However, it is unclear whether dimebon can exert disease-modifying activity *in vivo *and benefit AD neuropathology and/or clinical symptoms in animal models of AD.

In this study, we used the TgCRND8 AD mouse model to investigate the potential role of dimebon on AD type neuropathology and cognitive deterioration. TgCRND8 AD mice treated with a dose of dimebon equivalent to that used in the human clinical trials exhibited a trend of cognitive function improvement as reflected by observations from MWM test learning and probe trials. This marginal improvement in cognitive behavioral function was also seen in the strain-, age-, gender matched wild type mice. Our results indicate that dimebon might influence cognition through mechanism(s) independent of Aβ-mediated neuropathology. Evaluation of neuropathology in the brain of dimebon treated TgCRND8 mice showed that dimebon had no effect on contents of total Aβ or soluble oligomeric Aβ in the brain, further supporting the argument that dimebon does not directly target amyloid precursor protein (APP) processing or Aβ oligomerization which has been proposed to be responsible for cognitive dysfunction in AD [21-26]. Our *in vitro *study using primary neuron culture also confirms that dimebon does not interfere with APP processing as reflected by the lack of alteration in the level of extracellular Aβ peptides concentration in the conditioned medium upon dimebon treatment. It was recently reported that acute dimebon treatment increased Aβ secretion in Swedish mutant APP-overexpressing N2a cells [27]. This apparent discrepancy from our studies could be due to the differences in the drug concentration, cell type and treatment duration. *In vitro *aggregation assay also revealed that dimebon does not directly interfere with Aβ aggregation. Collectively, our studies suggest that APP processing or Aβ oligomerization is not a direct target for the action of dimebon.

Energy metabolism has been proposed as one of the potential mechanisms for dimebon's action. For example, in vitro evidence demonstrated dimebon treatment protects primary neuron cultures against Aβ-mediated neurotoxicity [18,19]. There is also evidence dimebon protects primary neuron cultures against stress-induced mitochondrial dysfunction [20]. Nonetheless, recent evidence suggests dimebon-mediated neuroprotection might be independents of the drug's potential effects on energy metabolism. Indeed, Wu et al. [4] demonstrated dimebon at low concentrations, does not protect against glutamate-induced neurotoxicity in primary striatal neuronal culture. Consistent with the latter observation, we found chronic dimebon treatment did not significantly modulate global energy metabolism in the brain, as reflected by the lack of alteration in key enzymes activities, including citrate synthase which is the rate limiting enzyme in TCA cycle. While an increase in MDH activity has been reported in AD brains [28], we are yet to determine whether the decrease we observed in MDH activity following dimebon treatment is of direct beneficial effect in AD. It is also possible that dimebon might specifically influence the mitochondial function in select cell types, such as neurons, glial cells or astrocytes which were not addressed in this communication since we did not measure the enzyme activities at cellular levels.

Our observation that dimebon treatment marginally improved cognitive behavioral function in wild type mice suggests that dimebon might modulate cognitive function via mechanisms independent of Aβ-mechanism. Our finding is consistent with previous reports that dimebon improves cognition in novel object recognition test and short-term social recognition memory in wild type rats [1,29,30]. Dimebon is a drug that can interact with multiple targets with high affinity, including α-adrenergic receptors, histamine receptors and serotonin receptors [4,29]. It is also possible that the beneficial effect of dimebon on cognitive function in our study could be due to its inhibition of serotonin 5-HT6 receptors as proposed by Schaffhauser et al [29]. Further mechanistic studies will be necessary to elucidate the role of dimebon in cognition.

## Conclusion

In conclusion, our preclinical studies demonstrated that dimebon might have some beneficial effect in improving cognitive function independent of Alzheimer's disease-type Aβ-related mechanisms or global energy metabolism in the brain of the TgCRND8 AD mouse model. Observations from our studies as well as others suggest dimebon might improve cognition in wild type mice and rats through Aβ-independent mechanisms raise the possibility that dimebon might be able to benefit cognitive function in patients with other neurodegenerative disorders, such as HD, or in the aging population. Additional studies will be necessary to clarify the mechanisms by which dimebon might directly or indirectly benefit cognitive function.

## List of Abbreviations

Aβ: βamyloid; AD: Alzheimer's disease; APP: amyloid precursor protein; CS: Citrate synthase; DTNB: 5, 5'-dithiobis (2-nitrobenzoic acid); ESI: electrospray ionization; HD: Huntington's disease; HFIP: 1,1,1,3,3,3,-hexafluoro-2-propanol; KGDHC: Alpha-ketoglutarate dehydrogenase complex; LC-MS: liquid chromatography-mass spectrometry; MDH: malate dehydrogenase; MWM: Morris water maze; SIR: Selected Ion Response; TCA: tricarboxylic acid;

## Competing interests

The authors declare that they have no competing interests.

## Authors' contributions

JW participated in the design, execution and data analysis of all the experiments as well as participated in the manuscript writing; MGF performed the pharmacokinetic studies and manuscript writing. MV, MX and WZ performed the TCA cycle enzyme activity studies and in vitro studies. XQ and AC conducted animal behavior studies. LH and GMP participated in the experimental design, data analysis and manuscript writing. All authors read and approved the final manuscript.
